# Draft genome sequences of *Kluyveromyces marxianus* strain U848 isolated from *Agave sisalana*

**DOI:** 10.1128/mra.01173-24

**Published:** 2024-12-27

**Authors:** Egor A. Vasyagin, Alexey V. Beletsky, Maxim Yu. Shalamitskiy, Karina A. Semenova, Nikolai V. Ravin, Andrey V. Mardanov

**Affiliations:** 1 Institute of Bioengineering, Research Center of Biotechnology of the Russian Academy of Sciences, Moscow, Russia; 2 Research Institute of Viticulture and Winemaking “Magarach” of the Russian Academy of Sciences, Yalta, Russia; University of California Riverside, Riverside, California, USA

**Keywords:** *Kluyveromyces marxianus*, draft genome, winemaking

## Abstract

*Kluyveromyces marxianus* is a yeast widely used in the dairy industry and frequently isolated from vineyards and wineries. Its capacity to metabolize diverse sugars makes it highly promising for winemaking applications. We report the draft genome sequence of the *K. marxianus* strain U848.

## ANNOUNCEMENT

*Kluyveromyces marxianus* exhibits several advantages over *Saccharomyces cerevisiae* in terms of thermotolerance and the ability to metabolize lactose and other sugars producing ethanol (1). Moreover, unlike *S. cerevisiae*, *K. marxianus* exhibits significant pectinase activity due to its ability to produce extracellular endopolygalacturonase (2). This property is utilized in winemaking for the prefermentative cold maceration of grapes (3). Moreover, the use of *K. marxianus* enhances the color intensity and hue of the wine and increases the extraction of free-run juice, improving the aromatic quality of wines (4).

We present the draft genome sequence of the *K. marxianus* strain U848, isolated from rotting leaf of *Agave sisalana*. The strain was obtained from the All-Russian collection of microorganisms and grown in YPD medium at 30°C. Genomic DNA was extracted using DNeasy PowerSoil DNA Isolation Kit (Qiagen, Germany) following the manufacturer’s protocol. Sequencing of the NEBNext Ultra II DNA Library (NEB, Ipswich, USA) on an Illumina MiSeq instrument with the MiSeq Reagent Kit v.3 (Illumina Inc., San Diego, CA, USA) yielded 1,028,609 read pairs (2 × 300 bp). Adapter sequences were removed using Cutadapt v.1.8 (5), overlapping paired reads were merged using FLASH (6), low-quality read ends were trimmed by Sickle v.1.33 (7) with q = 30 threshold. Illumina reads were assembled using SPAdes v.3.15.4 (8) with an “-isolate” parameter.

Gene structures were predicted by Augustus v.3.4.0 (9). BLAT v.34 (10) splice-aware alignments of *K. marxianus* strain DMKU3-1042 (GCF_001417885.1) (11) genes to contigs were incorporated into Augustus workflow as hints to improve intron and exon predictions.

The top 20 best hits in the Uniref database (12) were identified for the predicted genes by Diamond v.2.0.13 (13) BLASTP search using 1e−10 e-value cutoff. The best hit with the assigned function was used for annotation. rRNA genes were predicted by Barrnap v.0.9 (14) with a “–kingdom euk” parameter. tRNA genes were predicted by tRNAscan-SE v.2.0.9 (15). Genome completeness was assessed using BUSCO v.5.7.1 (16) with *Saccharomycetes* database. One contig, 35,513 bp in length, represented the mitochondrial genome. Genome statistics are described in Table 1. Taxonomic assignment based on 33 complete genomes from the NCBI database (accessed 27 Sep 2024) showed that the closest genome belongs to *K. marxianus* strain Olga-1 (GCA_016584275.1), isolated from kefir, with an average nucleotide sequence identity of 99.14%, calculated by FastANI v.1.33 (17). Default parameters were used for all software unless otherwise specified.

**TABLE 1:** Genome sequencing statistics

Parameter	Value
Illumina sequencing	
Reads number	2,057,218
Total bases (nt)	619,222,618
Genome assembly	
Total length	10,796,506 bp
Number of contigs	279
The longest contig	508,291 bp
N50	139,547 bp
GC content (%)	40.05
Protein-coding genes	4,696
tRNA genes	178
Genome coverage	31×
Copies of rRNA gene cluster	About 42
Genome assembly assessment	
Complete BUSCOs	95.6%
Fragmented BUSCOs	1.1%
Missing BUSCOs	3.3%

These genomic data will be useful for comparative genomics analysis of *K. marxianus* and its application in the food industry.

## Data Availability

The annotated draft genome of *Kluyveromyces marxianus* strain U848 has been deposited in DDBJ/ENA/GenBank under the accession number JBIHLH000000000, and raw reads are available at the NCBI Sequence Read Archive under accession number SRR30683221. The version described in this paper is the first version.
